# *Pinus radiata* genome reveals a downward demographic trajectory and opportunities for genomics-assisted breeding

**DOI:** 10.1093/g3journal/jkaf125

**Published:** 2025-06-05

**Authors:** Shane Sturrock, Tancred Frickey, Jules Freeman, Jakob Butler, Steffi Fritsche, Paloma Gea, Natalie Graham, Lucy Macdonald, Céline Mercier, Mark Paget, Leonardo Rippel Salgado, Frances Sussmilch, Emily Telfer, Phillip Wilcox, Heidi Dungey, Gancho Slavov

**Affiliations:** Computational Science, Institute of Environment Science and Research Ltd, 120 Mt Albert Road, Sandringham, Auckland 1025, New Zealand; Scion, Te Papa Tipu Innovation Park, Tītokorangi Drive, Rotorua 3010, New Zealand; Scion, Te Papa Tipu Innovation Park, Tītokorangi Drive, Rotorua 3010, New Zealand; Scion, Te Papa Tipu Innovation Park, Tītokorangi Drive, Rotorua 3010, New Zealand; School of Natural Sciences, and ARC Training Centre for Forest Value, University of Tasmania, Churchill Avenue, Hobart, TAS 7005, Australia; School of Natural Sciences, and ARC Centre for Plant Success in Nature and Agriculture, University of Tasmania, Churchill Avenue, Hobart, TAS 7005, Australia; Scion, Te Papa Tipu Innovation Park, Tītokorangi Drive, Rotorua 3010, New Zealand; Scion, Te Papa Tipu Innovation Park, Tītokorangi Drive, Rotorua 3010, New Zealand; Institute of Microbiology, University of Innsbruck, Technikerstrasse 25d, Innsbruck A-6020, Austria; Scion, Te Papa Tipu Innovation Park, Tītokorangi Drive, Rotorua 3010, New Zealand; Scion, Te Papa Tipu Innovation Park, Tītokorangi Drive, Rotorua 3010, New Zealand; Scion, Te Papa Tipu Innovation Park, Tītokorangi Drive, Rotorua 3010, New Zealand; Radiata Pine Breeding Company, 99 Sala Street, Rotorua 3010, New Zealand; Scion, Te Papa Tipu Innovation Park, Tītokorangi Drive, Rotorua 3010, New Zealand; Plant and Food Research, Dept. of Breeding and Genomics, Te Puke 3182, New Zealand; School of Natural Sciences, and ARC Centre for Plant Success in Nature and Agriculture, University of Tasmania, Churchill Avenue, Hobart, TAS 7005, Australia; Scion, Te Papa Tipu Innovation Park, Tītokorangi Drive, Rotorua 3010, New Zealand; Forestry Insights, Te Uru Rākau-New Zealand Forest Service, Ministry for Primary Industries, Te Papa Tipu Innovation Park, Sala Street, Rotorua 3010, New Zealand; Scion, Te Papa Tipu Innovation Park, Tītokorangi Drive, Rotorua 3010, New Zealand; University of Otago, Dept. Mathematics & Statistics, Dunedin 9016, New Zealand; Scion, Te Papa Tipu Innovation Park, Tītokorangi Drive, Rotorua 3010, New Zealand; PF Olsen Ltd, 49 Sala Street, Rotorua 3010, New Zealand; Scion, Te Papa Tipu Innovation Park, Tītokorangi Drive, Rotorua 3010, New Zealand; Radiata Pine Breeding Company, 99 Sala Street, Rotorua 3010, New Zealand; Institute of Biological, Environmental and Rural Sciences, Aberystwyth University, SY23 3EE, UK

**Keywords:** *Pinus radiata*, radiata pine, conifer, linkage disequilibrium, GWAS, genome assembly

## Abstract

*Pinus radiata* D. Don is one of the most widely planted exotic conifers. It is also a threatened species because native populations are small, disjunct, and challenged by pests and pathogens, deforestation, and climate maladaptation. Genomic tools can both enhance genetic improvement in operational breeding programs, and support conservation efforts. Using PacBio long-read sequencing, we assembled 20.6 Gbp of the large and complex *P. radiata* genome into 305,330 scaffolds, achieving a scaffold N50 of 196.22 kbp, which corresponds to 89% of its estimated genome size. Gene annotation, based on transcriptome data with a 97.9% BUSCO score, yielded 86,039 gene models. Linkage maps were used to anchor 7,952 contigs totaling 1.79 Gbp (approximately 9% of the assembly) across 12 pseudomolecules, which included c. 26% of the predicted genes. Genome resequencing (5.2×) of 40 trees, from 4 native populations and a major Australasian breeding population, uncovered c. 608.3 M SNPs which were used for population genomic analyses. A key finding of these analyses was the slower-than-expected decay of linkage disequilibrium (i.e. *r*^2^ > 0.2 up to 30 kb for SNPs with minor allele frequencies ≥ 0.10), suggesting recent drastic reductions of effective population size. Our findings indicate that genomic prediction could use fewer markers than the ca. 30 k that are currently employed. Additionally, this study highlights the potential for Southern Hemisphere breeding programs as ex situ conservation resources and established a foundation for functional characterization of the *P. radiata* genome.

## Introduction

The *Pinus* subsection *Australes* (Louden) comprises ca. 30 species of great ecological and economic importance (Gernandt *et al*. 2018), including *Pinus radiata* D. Don*. Pinus radiata* is one of the most widely planted exotic conifers, yet it is also on the World List of Threatened Trees (Mead 2013). Natural populations are small and disjunct (Gapare 2014), restricted to 3 locations in Central California, and on 2 Mexican Islands, Guadalupe and Cedros, off Baja California. The geographically isolated island populations are morphologically and genetically distinct, and classified differently to the type variety (*P. radiata* var. *binata* on Guadalupe and *P. radiata* var. *cedrosensis* on Cedros) (Karhu *et al*. 2006). Fossil evidence suggests that native populations have been dramatically reduced by climate change in recent millennia (Millar 1999). More recently, land clearance has further reduced and fragmented *P. radiata* habitats, leading to a decline in health in most stands, and native populations are under serious threat by pine pitch canker (*Fusarium circinatum*) (Reynolds *et al*. 2019).

*Pinus radiata* was introduced to New Zealand and Australia in the mid-19th century and is now widely grown in these and other countries, including Chile and Spain. New Zealand breeding efforts started in the early 1950s (see recent review (Paget 2022) and references therein) by phenotypic selection of “plus trees” with superior growth and form from local landrace populations, which were assumed to be mostly of Año Nuevo and Monterey ancestry (Burdon *et al*. 1997). Subsequent analysis of population structure in New Zealand *P. radiata* breeding populations has shown that island provenance ancestry is more common than previously thought, and a large proportion of the diversity from native provenances is likely captured in the current breeding program (Graham *et al*. 2022). A breeding program for New Zealand and parts of Australia is run by The Radiata Pine Breeding Company (RPBC) and is now in its third generation. While the initial focus was on growth and form (Dungey *et al*. 2009), genetically improved material is now available for growth, form, wood density, and stiffness (Paget 2022).

The development of genomic resources for *P. radiata* will facilitate effective extraction of the best genotypes from the breeding program for planting, as well as conservation outcomes. Genetic improvement in *P. radiata* has been very effective to date (Paget 2022), but genomic tools will accelerate improvement (Grattapaglia 2022; Paget 2022) and provide agility to respond to climate change and market uncertainty (Rogers 2004). Genomic selection is being implemented operationally by the RPBC, with 5,000–10,000 seedlings genotyped per year, and promises to deliver genetic gain more rapidly (Klápště *et al*. 2022; Paget 2022). Genomic resources not only underpin improvements in marker selection but also improve genomic prediction and enable functional characterization of the genome for potential biotechnological modification (Strauss *et al*. 2022).

We present the first genome assembly, annotation, and resequencing data for *P. radiata*, with the latter using genotypes selected to represent known diversity across the RPBC's New Zealand breeding program and the natural range of *P. radiata*. We illustrate how these resources will affect species conservation and improve genomic selection efficiency in operational breeding programs (Klápště *et al*. 2022).

## Materials and methods

### Sample selection and sequencing

Tree 268345 was selected for genome assembly, as it is an important parent/grandparent in the RPBC breeding program. This genotype was also used for the development of transcriptome resources (Telfer *et al*. 2018), marker panels (Graham *et al*. 2022), and high-density linkage mapping (Freeman *et al*. 2022).

#### Illumina HiSeq paired-end and mate-pair sequencing

Haploid megagametophyte tissue was excised from 18 seeds, and DNA extracted from each using a modified CTAB method (Telfer *et al*. 2013). Samples were purified using Zymo's Genomic DNA Clean and Concentrator kit (Zymo Research Corporation, Irvine, CA, USA), and DNA eluted in 50 µL of 10 mM Tris–HCl (pH 8.0). After quantification and purity assessment, an additional purification step using Qiagen's QIAquick PCR Purification kit (Qiagen, Düsseldorf, GER) was performed with elution in 60 μL of EB buffer. Haploid status and congruity with the previously determined marker profile for 268345 was confirmed using a set of 6 *P. radiata* simple sequence repeat (SSR) markers (data not shown). Megagametophyte extractions were supplied to New Zealand Genomics Ltd (NZGL, Dunedin, New Zealand), with 8 selected for preparing Illumina TruSeq (Illumina, Carlsbad, CA, USA) libraries with an average insert size of 500–550 bp. Libraries were pooled and run on a single lane of HiSeq 2000 and assessed for complexity, whereafter the best library was selected for further sequencing on 20 lanes of HiSeq 2000 (2 × 100 bp paired-end) and 20 lanes of HiSeq v4 (2 × 125 bp paired-end). In addition, 150 mg of diploid needle tissue was used in each of 6 further DNA extractions for Illumina mate-pair reads. Tissue was ground to a fine powder under liquid nitrogen using a mortar and pestle, and DNA extracted using the NucleoSpin Plant II (Machery-Nagel, Düren, GER) kit as described by Telfer *et al*. (2013). Replicates were combined and supplied to NZGL for library preparation. Libraries with insert sizes of 3, 5, and 10 kb were prepared.

#### PacBio long-read sequencing

To generate PacBio long reads, high molecular weight DNA was extracted from a range of tissues (including meristematic bud tissue, needle tissue, and male catkins), with the NucleoSpin Plant II kit, as described by Telfer *et al*. (2013), using 250 mg of tissue per sample ground to a fine power under liquid nitrogen. Additional precipitations were performed by adding 1/2 volume of 1.9 M ammonium acetate and 1 volume isopropanol (room temperature) and mixing by gentle inversion for 10 min. After centrifugation for 30 min at 11,000 × *g* at 4°C, the supernatant was decanted, the DNA pellet washed in room temperature 70% (v/v) ethanol, and air-dried before resuspension in 1× TE buffer pH 8.0. Samples were then pooled, mixed 4:1 with DNAstable Plus (Biomatrica, San Diego, CA, USA), and shipped on frozen ice-packs to the Ramaciotti Centre for Genomics (University of New South Wales, Sydney, Australia) for sequencing on the PacBio (Pacific Biosciences, Menlo Park, CA, USA) SMRT sequencing platform (Korlach 2012). An additional AMpure bead purification (Beckman Coulter Inc., Brea, CA, USA) was performed, and sample integrity assessed using the 10–48 kb protocol on a Pippin Pulse electrophoresis gel (Sage Science, Beverly, MA, USA). SMRTbell (Pacific Biosciences, Menlo Park, USA) libraries with 10–20 kb insert sizes were prepared (without additional shearing) and run on the PacBio Sequel Sequencer. To improve coverage, a second round of PacBio sequencing was also undertaken, with slightly different methods (see Supplementary Methods).

#### RNA sequencing

Transcriptome datasets were generated from a range of *P. radiata* tissues at different developmental (from freshly germinated seed, embling and callus in tissue culture, to mature trees) and temporal stages (Supplementary Table 4), to aid identification of genes and genome annotation. Tissues (∼100 mg) were ground to a fine powder under liquid nitrogen and RNA extracted using the Spectrum RNA extraction kit (Sigma-Aldrich Co. LLC, St. Louis, USA) following the manufacturer's protocol. RNA was shipped Custom Science Ltd. (Auckland, New Zealand) using the RNAstable buffer from Biomatrica. The samples were sequenced using PacBio Sequel II and yielded 6,456,255 reads (16.6 Gbp) in total.

### Genome assembly and scaffolding

tAssembly of the PacBio sequencing data was performed using the wtdbg2 assembler (Ruan and Li 2020), with the following settings: “-t 64 -x rs -p 21 -S 2 --aln-noskip --rescue-low-cov-edges --tidy-reads 5000 -l5120”. When running wtdbg2, a large overlap of 5 kbp was chosen to limit the potential for repeats collapsing. Using long reads, minimap2 (Li 2016) was used to polish the assemblies, and BWA-MEM (Li 2013) used for the final error correction with Illumina short reads. Initial scaffolding using the 3, 5, and 10 kb mate pair libraries was performed using BESST (Sahlin *et al*. 2014).

*Pinus radiata* linkage maps (Freeman *et al*. 2022) were used to order and orient as many contigs as possible into 12 pseudomolecules. This scaffolding was performed using the software package ALLMAPS (Tang *et al*. 2015) and 4 parental linkage maps (268405, 268345, 850055, 850096), excluding linkage group 8 from the 850096 parent due to a putative inter-chromosomal rearrangement in this linkage group relative to all other parents (Freeman *et al*. 2022). One marker that mapped to more than 1 linkage group was also excluded, resulting in 14,035 markers used in ALLMAPS. Marker sequences were mapped to contigs via BWA-MEM using default settings. In the case of identical hits to multiple contigs, the largest contig that had not already been matched to a marker was chosen, except in cases of adjacent markers suggesting a smaller contig spanning multiple markers. The following weighting was chosen to maximize sequence ordered and oriented within ALLMAPS: weight 3 (highest) for parent 268345, weight 2 for parent 268405, and weight 1 for the other 2 parents.

Before assembling into pseudomolecules, ALLMAPS was used to split potential chimeric contigs (contigs assigned to multiple markers originating in different linkage groups) where there was evidence from at least 2 markers on either side of a proposed split (allmaps split –chunk = 2). Splits were localized to the largest region of Ns between markers, or directly after the final marker of the first chimeric section if no Ns were present. Subsequently, transcript evidence was used to join additional contigs, by mapping transcripts on to the genome contigs via BWA-MEM using default parameters, and filtering by quality and coverage, with only transcript hits in a compatible orientation with 80% transcript sequence matched to contigs and a maximum of 50% overlap across different contigs retained. The most abundant joining was chosen in cases of conflicts between sequence overlaps. This approach resulted in 25,132 contigs being joined into 11,833 longer contigs. The new joined contigs were then tested for chimerism and split again, and finally, all mapped contigs were assembled into pseudomolecules using ALLMAPS.

### Repetitive element analysis and gene annotation

The genome was filtered for repeat elements using EDTA v2.0.0 with default settings (Su *et al*. 2021). Due to the size of the genome and resulting compute and memory limitations, the genome (305,388 contigs) was split into smaller sets of 10,000 sequences, and the results pooled across all sets. To compare the repetitive content of *P. radiata* and *Pinus taeda* (Supplementary Table 3), a TBLASTN search with the 100 amino acid long reverse transcriptase (RT) sequence for both Gypsy and Copia elements was performed on each assembly, with a cutoff at 80% query coverage. The paralogous RT sequences from both species for each superfamily were aligned using MUSCLE (Edgar 2004).

The 24 transcriptomics datasets (Supplementary Table 4) were combined and processed using Trinity v2.9.1 (Haas *et al*. 2013) with default settings. The resulting set of 68,534 assembled and deduplicated transcripts were analyzed using BUSCO v5.3.2 (Simão *et al*. 2015) (‘-m geno -l viridiplantae_odb10'). GMAP v2021-12-17 (Wu and Watanabe 2005) was used to create a GFF3 file providing the putative location of the Trinity processed transcripts in the repeat-masked genome. In addition, the Trinity processed transcripts were translated in 6 open reading frames (ORF), the longest ORF for each was selected and PhyloGenie (Frickey and Lupas 2004) was used to search public databases for similar protein sequences, generate multiple sequence alignments, and infer phylogenetic trees for each of the transcripts. A putative transitive functional annotation was derived by post-processing the generated files while giving preference to annotations derived from orthologs and curated databases over other sources. The annotation data is available as a Supplementary material file.

### Resequencing and population genomics

Forty trees were selected to represent genetic diversity across the RPBC's breeding program and the natural range of *P. radiata*. Genomic DNA was extracted from either needle or cambium tissue by Slipstream Automation (Palmerston North, New Zealand), using proprietary CTAB-based methods. DNA was quantified as described by Telfer *et al*. (2019). Resequencing was then performed by Rapid Genomics (Gainesville, Florida, USA) using Illumina PE150 to generate at least 120 Gbp (∼5.2× coverage) of sequence for each sample.

After quality filtering with Fastp (v0.21.0) (Chen *et al*. 2018), reads were mapped to the *P. radiata* reference genome V1, using BWA-MEM with “-a” and “-M” options for compatibility with subsequent tools. Mapped reads were sorted using SAMtools sort (v1.12) (Li *et al*. 2009; Danecek *et al*. 2021), and deduplicated using Picard MarkDuplicates (v2.26.11, http://broadinstitute.github.io/picard/), with options “-REMOVE_DUPLICATES TRUE” and “-CREATE_INDEX TRUE”. To accelerate analyses, the resulting BAM files were trimmed using SAMtools with options “-h”, “-b”, and “-L” to only include read alignments to the 62,433 scaffolds that were at least 100 kb in length. Trimmed BAM files were then indexed using SAMtools, and used for SNP discovery and genotype calling using DeepVariant (v1.1.0) (Poplin *et al*. 2018), ultimately resulting in a single gVCF file for each re-sequenced individual. Individual gVCF files were merged using GLNexus (v1.2.7) (Lin *et al*. 2018) with options “--config DeepVariant_unfiltered” and “--trim-uncalled-alleles”, into a BCF file containing 660,611,945 polymorphic sites. Finally, the BCF file was converted to VCF using BCFtools view (v1.12) (Danecek *et al*. 2021).

Filtering for SNP quality score (Q ≥ 30), deviations from Hardy–Weinberg equilibrium (*P* > 0.0001), and missing data (≤20%) was performed using VCFtools v. 0.1.16 (Danecek *et al*. 2011). Density of segregating sites, nucleotide diversity, and Tajima's *D* were calculated in 10 kb windows using VCFtools. Principal component analysis (PCA) and linkage disequilibrium (LD) analyses were performed using PLINK v1.9 (Chang *et al*. 2015). LD was calculated using pairs of SNPs within windows of 100 kb, using minor allele frequency (MAF) thresholds of 0.05 and 0.10. Average *r*^2^ value for SNPs within 1 kb bins was calculated and plotted for each SNP filtering set, with LD extent defined as the distance where *r*^2^ = decayed to 0.2. For PCA, 1 million SNPs with MAF > 0.05 were first selected randomly and then pruned to eliminate 1 SNP from each pair of SNPs that were located within 100 kb of each other and were in LD (*r*^2^ > 0.2), resulting in 739,332 “independent” SNPs. Estimates of effective population size from the same set of SNPs were obtained using SNeP (Barbato *et al*. 2015), assuming *c* = 10^−9^ and a = 2.2 (Corbin *et al*. 2012), and visualized as heatmap-like scatter-plots generated using the heatscatter function of the LSD R package (Schwalb *et al*. 2020). In addition, trend lines were estimated as rolling medians of 11-datapoint windows (*rollmedian* function of the zoo R package (Zeileis and Grothendieck 2005)) and were then denoised using the *smooth.spline* R function.

### Identification of genes linked to SNP-trait associations identified by genome-wide association study (GWAS)

A genome-wide association study (GWAS) was performed using phenotypes expressed as estimated breeding values (EBVs) for 4,564–15,052 trees from the RPBC breeding program. Depending on the trait, EBVs were calculated based on phenotypic measurements for 115,170–441,936 trees from 60–117 breeding field trials using the BOLT software (Theta Solutions LLC, 2021; Paget 2022). We performed GWAS for the following traits: diameter at breast height (DBH, GWAS *N* = 15,052); branching frequency (BRA, GWAS *N* = 15,051); straightness (STR, GWAS *N* = 15,052); wood density (DEN, GWAS *N* = 11,874); predicted modulus of elasticity (PME, GWAS *N* = 11,816); and Dothistroma needle blight severity (DOTHI.Y, GWAS *N* = 4,564). The genotype data for GWAS was generated using the Axiom SNP array for *P. radiata* (Graham *et al*. 2022), which yielded 36,285 SNPs across the genome. The GWAS analysis was performed using EMMAX (Kang *et al*. 2010), with default options for univariate analysis and incorporating both an identity-by-state relatedness matrix and 5 principal components of the genotype data to account for population structure and relatedness within the breeding populations. Associations with *P* < 5e^−05^ were considered significant. Identification of candidate genes, within the scaffolds harboring the 41 significant SNP-trait associations with DBH, was attempted. When LD to 30 kb exceeded 0.2 for one of these scaffolds, we also investigated the neighboring scaffolds.

The scaffold sequences (or a region ∼100–120 kb for very large scaffolds) were used as queries with NCBI's protein BLAST suite (https://blast.ncbi.nlm.nih.gov/Blast.cgi) or TAIR's BLAST search (https://www.arabidopsis.org/, TAIR BLAST 2.9.0+) against Arabidopsis proteins (Araport11; Cheng *et al*. 2017) either directly (BLASTx) or after derived protein sequences were predicted for *P. radiata* (BLASTp) using the HMM gene structure prediction software of the Softberry online tool (Solovyev *et al*. 2006). Genes of interest on scaffolds associated with DBH were selected based on characterized roles of homologs in other plant species. These included genes involved specifically in the control of secondary growth or controlling growth, cell division/expansion, cell wall composition, and/or stomatal behavior. Gene identity was confirmed by reciprocal tBLASTn searches of Arabidopsis protein hits against the *P. radiata* genome.

## Results and discussion

**Fig. 1.**
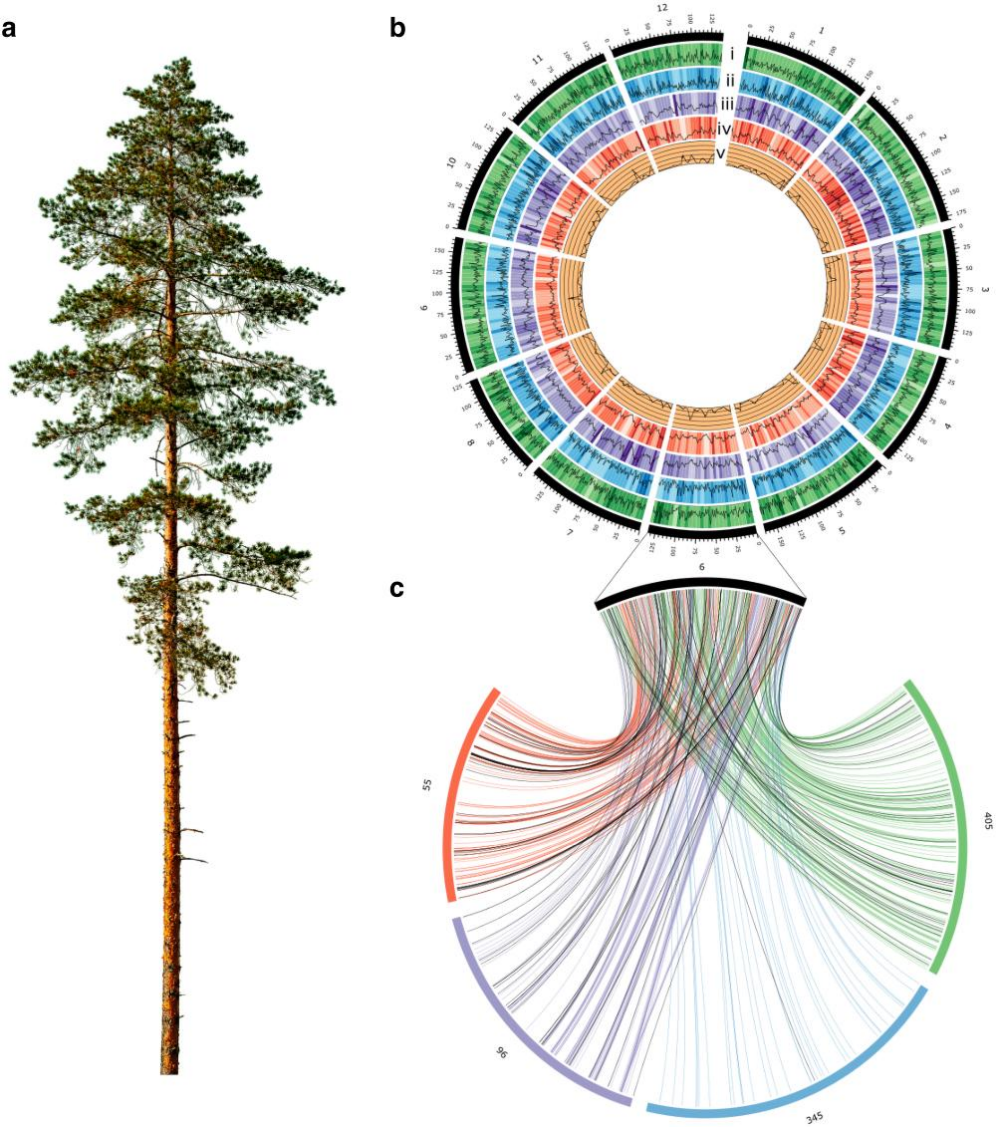
Genome assembly of *Pinus radiata.* a) Example of *P. radiata.* b) Genome features across 1 Mb (line) and 5 Mb (heatmap) windows: i) intact repeat density (0–15% per Mb, 0–9% per 5 Mb); ii) gene density (0–38 genes per Mb, 5–106 genes per 5 Mb); iii) nucleotide diversity (0.003–0.007 per Mb, 0.003–0.006 per 5 Mb); iv) LD-based effective population size (6,782–43,793 per Mb, 7,776–31,587 per 5 Mb); v) recombination rate (0–0.94 cm/Mb). c) Synteny of chromosome 6 with LG6 from 4 linkage maps. Black lines represent non-collinear markers.

We assembled 20.6 Gbp of the large, complex *P. radiata* genome, which is 89% of its estimated size (Zonneveld 2012 ) (Fig. 1b). Assembly contiguity was higher than that in the similarly sized *P. taeda* genome (Zimin *et al*. 2017), but lower than in *Pinus tabuliformis* (Niu *et al*. 2022) or *Pinus albicaulis* (Neale *et al*. 2024) (Supplementary Table 1). Integrating the *P. radiata* assembly with linkage maps, we anchored 7,952 contigs with 1.79 Gbp total length (approximately 9% of the assembly) across 12 pseudomolecules, presumably corresponding to chromosomes (Fig. 1b and c, Supplementary Tables 1 and 2). Marker order on pseudomolecules was highly collinear with individual parental linkage maps, with an average Spearman correlation of 0.82 (Fig. 1d, Supplementary Table 2).

Our initial estimates of 39% repetitive content across the *P. radiata* genome contrast with that of *P. taeda* (82% repeats) and *P. albicaulis* (77% repeats). Actual repeat content is likely to be higher than our estimate, primarily because we used a stringent minimum overlap of 5 kbp during sequence assembly to prevent repeat collapse. This allowed us to more accurately assess the length of assembled repeat regions, but likely contributed to a greater proportion of repeats left unassembled and therefore unassessed. Further analysis is needed to confirm this and fully characterize the likely much higher repetitive content of the *P. radiata* genome. Consistent with this, the proportion of intact repeat elements (10%) appears to be larger in *P. radiata* than in *P. taeda* (Neale *et al*. 2014), although this comparison is likely confounded by assembly status and annotation methods. For example, the assembly of *P. taeda* has nearly 10-fold more contigs which would contribute to fewer intact repeat elements (Supplementary Table 3). Regardless of these caveats, our phylogenetic analysis of intact long terminal repeat (LTR) elements was informative. Paralogues from LTR retrotransposons from *P. radiata* and *P. taeda* were present in all major phylogenetic branches, suggesting that expansion in the repetitive superfamilies occurred before these species diverged (Supplementary Figs. 1 and 2; Methods).

Gene annotation was supported using transcriptome data from 15 tissues (Supplementary Table 4). Assembly and deduplication yielded 68,193 transcripts with a 97.9% BUSCO score, indicating near-complete gene coverage. Mapping transcriptomes against a repeat-masked version of the genome resulted in 86,039 predicted genes, approximately 26% of which were present in the scaffolded pseudomolecules. The number of annotated genes in our assembly was similar to the 80,495 genes annotated in *P. tabuliformis* (Niu *et al*. 2022), but greater than the 50,172 genes annotated in *P. taeda* (Neale *et al*. 2014) or the 27,555 genes in *P. albicaulis* (Neale *et al*. 2024). Gene annotation in conifers is challenging due to their large genome sizes, very long introns (Niu *et al*. 2022), high repeat contents, abundant pseudogenes, and large gene families. The magnitude of differences in the number of annotated genes between *Pinus* assemblies likely reflects numerous factors, including the contiguity and coverage of each genome assembly and annotation techniques. Even within assemblies. different annotation and filtering techniques produce widely different numbers of annotated genes (Niu *et al*. 2022; Neale *et al*. 2024).

**Fig. 2.**
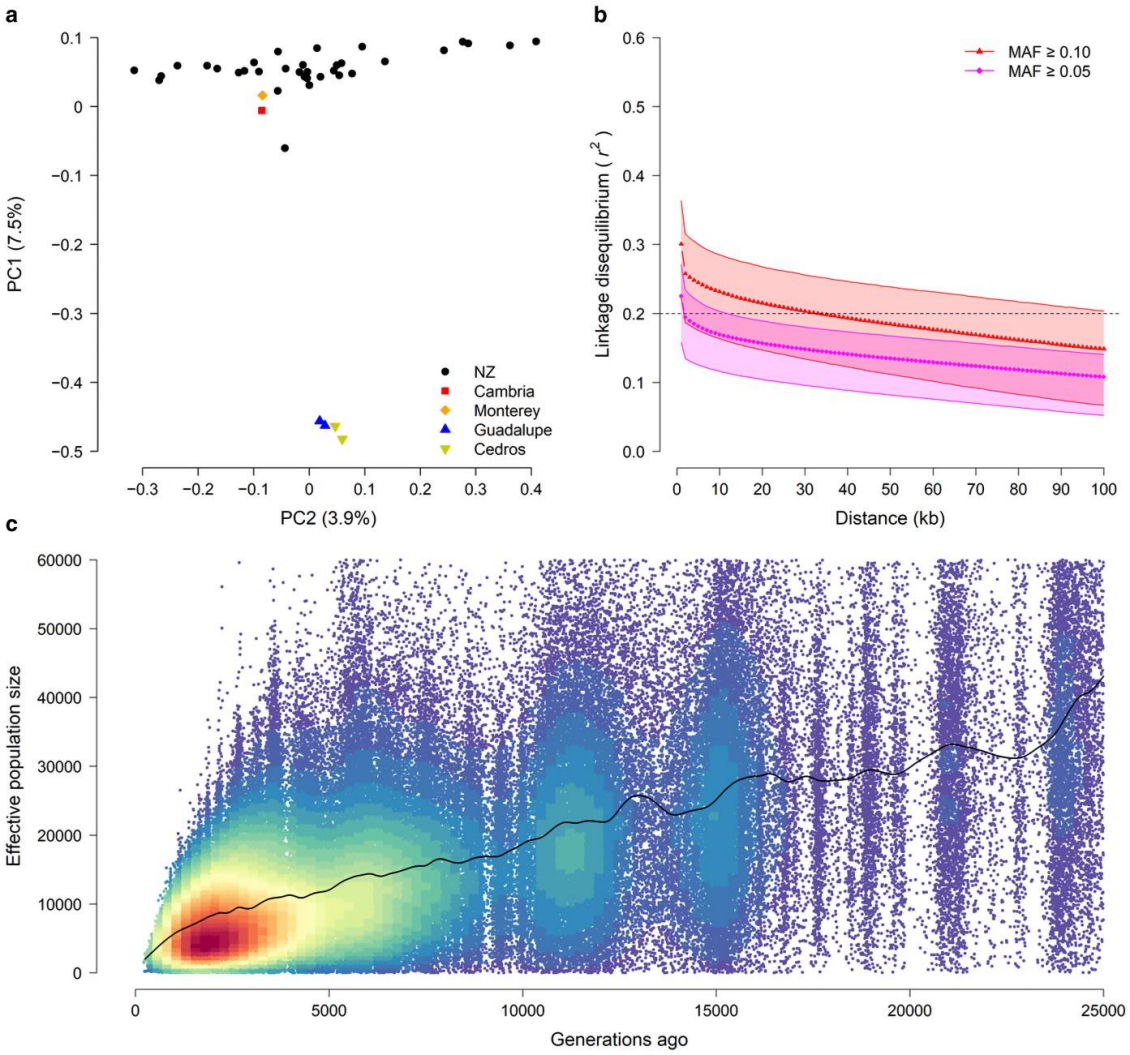
Population structure, linkage disequilibrium (LD), and recent (i.e. LD-based) effective population size of *P. radiata*. a) Principal component analysis of SNP data. Colors and symbols indicate native provenance origin; black circles represent the New Zealand breeding population. Numbers in parentheses correspond to percentage of variation explained by the first 2 principal components (PC1 and PC2). b) LD decay with physical distance among loci. Shaded areas delineate the different *r*^2^ interquartile ranges for SNPs with minor allele frequencies (MAF) ≥ 0.05 and 0.10. c) Trendline with estimates of LD-based effective population size, based on filtered SNPs with MAF > 0.05 (see Materials and methods).

Genome resequencing (∼5.2× coverage) of 40 *P. radiata* trees allowed the discovery of approximately 608.3 M SNPs and 52.3 M short indels (Supplementary Table 5). PCA confirmed the previously characterized population genetic structure of the New Zealand *P. radiata* breeding program (Fig. 2a, Graham *et al*. 2022). Genome-wide estimates of nucleotide diversity (*π* = 0.0035–0.0039, Supplementary Table 5) were similar to those of *P. taeda* (Eckert *et al*. 2013) and *Populus trichocarpa* (Evans *et al*. 2014), indicating that the historical effective population size of *P. radiata* was relatively large. This contrasts with previous hypotheses that molecular genetic diversity may be reduced in *P. radiata*, compared with other pine species (Moran *et al*. 1988; Hamrick *et al*. 1992). Similar to *P. taeda* (Eckert *et al*. 2013), estimates of Tajima's *D* tended to be negative, indicating an excess of rare variants, but varied considerably across the genome (Supplementary Table 5).

LD decayed slower than expected in *P. radiata* compared with other forest trees (Fig. 2b). For example, average *r*^2^ for common SNPs (MAF ≥ 0.10) decayed to 0.2 within ∼30 kb, more than 5 times slower than *P. trichocarpa* (Slavov *et al*. 2012). This translated to relatively low estimates of the scaled recombination rate (4*N_e_c* = 0.00008–0.00011, Supplementary Table 5) and LD-based effective population size (*N_e_*). Recent (i.e. LD-based) *N_e_* decreased linearly until 500–1,000 generations ago, after which the rate of decline accelerated drastically (Fig. 2c, Supplementary Fig. 3). This is consistent with fossil evidence suggesting that *P. radiata* abundance decreased dramatically ca. 4–8 ka due to climate warming and aridification (Millar 1999), a trend that is likely to continue (McDowell *et al*. 2016, 2020).

A GWAS across approximately 15 k trees of the breeding population uncovered 104 significant associations (*P* < 10^−5^) across the 6 traits measured. The total variance explained by the combined effects of the significant SNPs ranged from 2.6% for PME to 8% for DOTHI (Supplementary Table 6). Candidate genes implicated by associations with DBH were associated with various biological functions including lignin biosynthesis, auxin biosynthesis and transport, and abscisic acid transport (Supplementary Table 7). Other implicated genes included genes involved in auxin biosynthesis (*YUCCA*) and transport (*PIN*), which have been linked to secondary growth regulation in *Populus* (Supplementary Table 7). Close relationships among the trees used for GWAS (i.e. from a breeding population) may have affected these results as the genomic control inflation factor (*λ*_GC_) exceeded 1.15 even after including up to 5 principal components in EMMAX analyses. Nevertheless, our findings provide a glimpse into the potential of future GWASs.

This study has several important implications. First, the historical trajectory in LD-based *N_e_*, combined with projections of further climate changes, indicates that *P. radiata* is unlikely to persist in its native range. Breeding programs should therefore also be regarded as valuable ex situ conservation resources. Second, the extensive genome-wide LD we detected is consistent with previous empirical results, suggesting that genomic prediction in *P. radiata* may be sufficiently accurate with much fewer SNPs than on existing genotyping platforms (Graham *et al*. 2022; Klápště *et al*. 2022). This is also advantageous for imputation for GWAS. Finally, as our genome assembly and annotations evolve, so will the functional characterization of gene targets for potential manipulation via biotechnological approaches.

## Supplementary Material

jkaf125_Supplementary_Data

## Data Availability

Short and long read archives on which this assembly is based, as well as the genome assembly, assembled proteome and predicted genes, will be accessible via public databases (e.g. GenBank). The assembled genome is available from NCBI under accession number JBDLLP000000000. The genome sequencing datasets upon which this work is based have been deposited with NCBI as BioProject PRJNA1094976. Accession numbers: SAMN40923754, SAMN40904542, SAMN40876404, SAMN40874349, SAMN40709891. The dataset on which the reference transcriptome is based has been deposited with NCBI as BioProject PRJNA1098359. Accession numbers: SAMN40906064, SAMN40906063, SAMN40906062, SAMN40906061, SAMN40906060, SAMN40906059, SAMN40906058, SAMN40906057, SAMN40906056, SAMN40906055, SAMN40906054, SAMN40906053, SAMN40906052, SAMN40906051, SAMN40906050. Annotations are available on GSA FigShare at https://doi.org/10.25387/g3.29218712. Breeding values (EBVs) are available at www.rpbc.co.nz/categories/latest-breeding-values or on request from the RPBC. Supplemental material available at G3 online.
