# Supplementary material for: Pinus radiata genome reveals a downward demographic trajectory and opportunities for genomics-assisted breeding
Source: G3 (Bethesda). 2025 Jun 5;15(8):jkaf125. doi: 10.1093/g3journal/jkaf125 (PMC12341877; doi:10.1093/g3journal/jkaf125)
Supplement: jkaf125_Supplementary_Data [file jkaf125_supplementary_data.zip › Figure_S1_G3-2024-404909.docx]

**
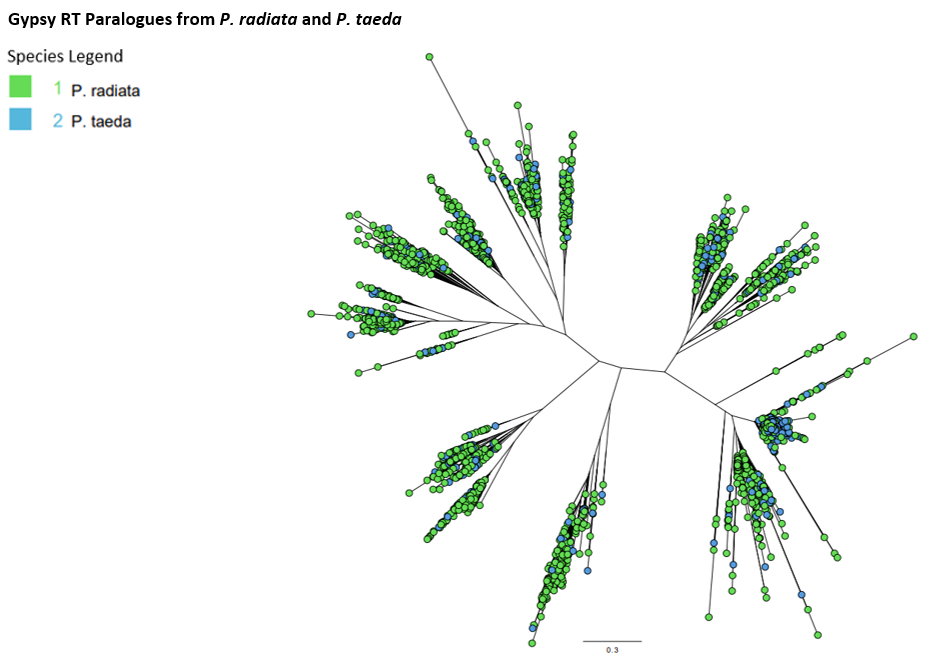
**

**Fig. S1** | Heuristic neighbor joining phylogenetic tree of the Gypsy RT paralogues identified in TBLASTN searches of *P. radiata* and *P. taeda*.
