# Supplementary material for: Pinus radiata genome reveals a downward demographic trajectory and opportunities for genomics-assisted breeding
Source: G3 (Bethesda). 2025 Jun 5;15(8):jkaf125. doi: 10.1093/g3journal/jkaf125 (PMC12341877; doi:10.1093/g3journal/jkaf125)
Supplement: jkaf125_Supplementary_Data [file jkaf125_supplementary_data.zip › Figure_S3_G3-2024-404909.docx]

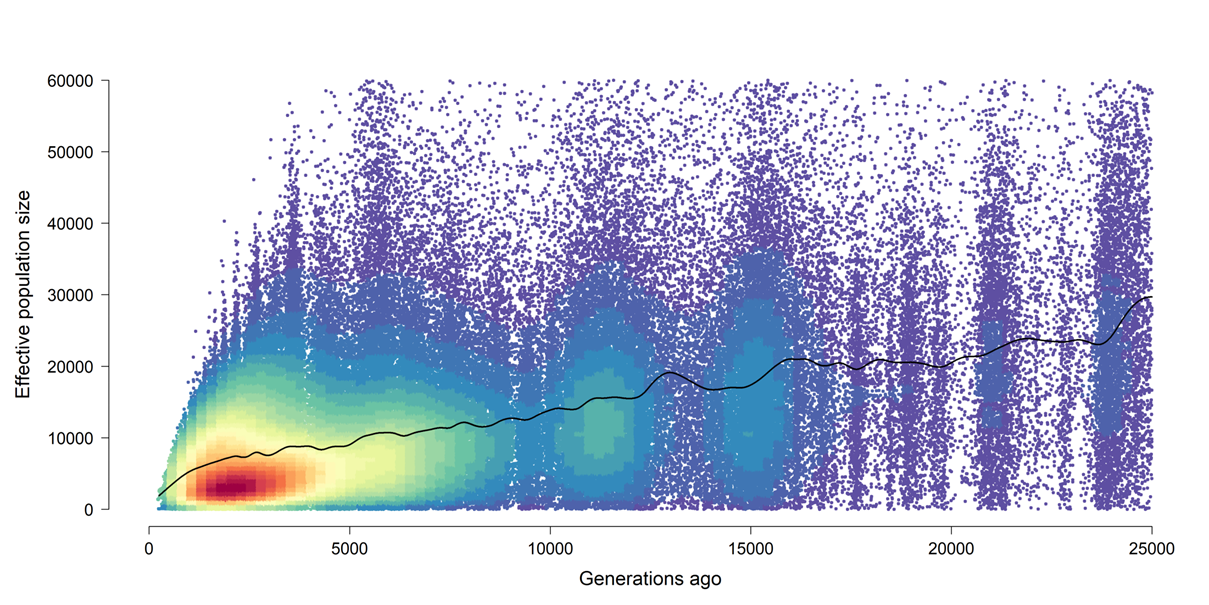


**Fig. S3 | Estimates of recent LD-based effective population size based on filtered SNPs with MAF > 0.10. The trendline is the spline-smoothed 11-point rolling median (see Methods).**
