# Supplementary material for: Pinus radiata genome reveals a downward demographic trajectory and opportunities for genomics-assisted breeding
Source: G3 (Bethesda). 2025 Jun 5;15(8):jkaf125. doi: 10.1093/g3journal/jkaf125 (PMC12341877; doi:10.1093/g3journal/jkaf125)
Supplement: jkaf125_Supplementary_Data [file jkaf125_supplementary_data.zip › Supplementary_Methods_G3-2024-404909.docx]

Supplementary methods

To improve the coverage of the long read data, a second round of PacBio sequencing was undertaken. This sequencing used DNA from the July 2017 needle collections performed from tree 268345 (described in the main Methods), using a CTAB-based protocol modified from Doyle and Doyle (1987) with two variations of the CTAB extraction buffer (A and B). Two replicates were performed for each buffer. Tissue (2 g) was homogenised with a pre-chilled mortar and pestle under liquid nitrogen. Pre-warmed (60°C) CTAB buffer (20 mL) (2% (w/v) CTAB, 1.4 M NaCl, 20 mM EDTA, 100 mM Tris HCl, 0.2% β-mercaptoethanol, 2% (w/v) PVP-40, 0.5 mg/ml proteinase K (extraction buffer A only)) was added and samples gently mixed by inversion. After 30 min incubation at 60°C with inversions every 10 min, samples were extracted with chloroform:isoamyl alcohol (24:1) (CIA). Cellular debris was pelleted by centrifugation for 15 min at 3,220 × g, and supernatant transferred to fresh tubes and 1× volume isopropanol (room temperature) added. After mixing by inversion and precipitation at -20°C for an hour, DNA was pelleted by centrifugation at 13,750 × g at 4°C for 30 min. DNA pellets were washed in 20 mL of 70% (v/v) ethanol (-20°C) and air-dried before resuspension overnight in 5 mL of 10 mM Tris HCl (pH 8.0). To each tube, RNase A was added to a final concentration of 100 μg/mL and incubated at 37°C for 60 min. Samples were transferred to 15 mL tubes, re-extracted with 1× volume CIA and centrifuged at 3,220 × g for 10 min. The aqueous phase was transferred to a fresh 50 mL tube and DNA precipitated with 1/10× volume 3 M sodium acetate (pH 5.2) and 2.5× volume of ethanol (-20°C), gentle mixing by inversion, and incubation at -20°C for 60 min. DNA was pelleted by centrifugation at 13,750 × g at 4*°*C for 40 min. Supernatant was discarded and pellets washed with 15 mL 70% (v/v) ethanol. Pellets were air-dried and resuspended for 2 days at 4°C in 1 mL (A samples) or 0.75 mL (B samples) of 10 mM Tris HCL (pH 8.0). Short incubations at 37*°*C were applied to facilitate pellet resuspension, when required. Concentration and absorbance ratios were determined as described in the main Methods, and DNA integrity confirmed through agarose gel electrophoresis. All four DNA samples were combined, gently mixed 4:1 with DNAstable® Plus and shipped on ice-packs to the Ramaciotti Centre for Genomics for sequencing on the PacBio Sequel platform, as described in the main Methods.

Doyle, J., & Doyle, J. (1987). A rapid DNA isolation procedure for small quantities of fresh leaf tissue. *Phytochemical Bulletin, 19*, 11-15.
