# Supplementary material for: Pinus radiata genome reveals a downward demographic trajectory and opportunities for genomics-assisted breeding
Source: G3 (Bethesda). 2025 Jun 5;15(8):jkaf125. doi: 10.1093/g3journal/jkaf125 (PMC12341877; doi:10.1093/g3journal/jkaf125)
Supplement: jkaf125_Supplementary_Data [file jkaf125_supplementary_data.zip › Table_S1_G3-2024-404909.docx]

**Table S1 Summary statistics for *Pinus* genome assemblies**

| **Assembly** | **N contigs** | **N pseudomolecules / scaffolds** | **Genome length** | **Contig N50** | **Scaffold N50** | **Annotated genes** | **BUSCO^a^** |
| --- | --- | --- | --- | --- | --- | --- | --- |
| ***P. radiata* wtdbg2^b^** | **329,668** | **0 / 305,330** | **20.62 Gbp** | **163.83 Kbp** | **196.22 Kbp** | 86,039 | S&D:69;F:27;M:4 |
| ***P. radiata* ALLMAPS^c^** | **7,952 (2.4%)** | **12 / 0** | **1.79 Gbp (8.7%)** | **340.58 Kbp** | **133.84 Mbp** | **25.9%^d^** | **S&D:27;F:17;M:57** |
| ***P. tabuliformis**** | **22,739** | **12 / 7,359** | **25.4 Gbp** | **2.60 Mbp** | **2.1 Gbp^e^** | **80,495** | **S&D:84;F:NA;M:NA** |
| ***P. taeda* MaSuRCA**** | **2,855,700** | 0 / 1,760,464 | **22.1 Gbp** | 25.36 Kbp | 107.04 Kbp | **50,172** | **NA** |
| ***P. albicaulis*†** | **92,740** | 12 / 34,176 | **27.6 Gbp** | 537.0 Kbp | 2.0 Gbp^f^ | **27,555** | **S&D:66;F19;M:15** |
| ***P. lambertiana*‡** | **14,950,590** | 0 / 4,253,097 | **25.5 Gbp** | 292.7 Kbp | 381.58 Kbp | **NA** | **NA** |

**^a^BUSCO – S: single copy, D: duplicated, F: fragmented, M: missing. Expressed as percentages. *P. radiata* assessed using BUSCO v5.3.2, *P. tabuliformis* assessed using BUSCO v4.1.4, *P. albicaulis* assessed using BUSCO v5.2.2. S&D combined to ensure comparability between studies.**

**^b^Statistics are based on the unmasked genome**

**^c^Statistics are based on the genome scaffolded into the 12 pseudomolecules. (%) shows percentage in the ALLMAPS assembly, relative to the entire wtdbg2 assembly.**

**^d^Estimated by percentage of transcripts mapping to pseudomolecules**

**^e^*P. tabuliformis* scaffold N50 based on 12 pseudomolecules**

**^f^*P. albicaulis* scaffold N50 based on 12 pseudomolecules**

***Niu et al. (2022).**

****Zimin et al. (2017)**

**†Neale et al. (2024)**

**‡Stevens et al. (2016)**

Neale, D. B., Zimin, A. V., Meltzer, A., Bhattarai, A., Amee, M., Figueroa Corona, L., . . . Wegrzyn, J. L. (2024). A genome sequence for the threatened whitebark pine. *G3 Genes|Genomes|Genetics, 14*(5). doi:10.1093/g3journal/jkae061

Niu, S., Li, J., Bo, W., Yang, W., Zuccolo, A., Giacomello, S., . . . Wu, H. X. (2022). The Chinese pine genome and methylome unveil key features of conifer evolution. *Cell, 185*(1), 204-217.e214. doi:<https://doi.org/10.1016/j.cell.2021.12.006>

Stevens, K. A., Wegrzyn, J. L., Zimin, A., Puiu, D., Crepeau, M., Cardeno, C., . . . Langley, C. H. (2016). Sequence of the Sugar Pine Megagenome. *Genetics, 204*(4), 1613-1626. doi:10.1534/genetics.116.193227

Zimin, A. V., Stevens, K. A., Crepeau, M. W., Puiu, D., Wegrzyn, J. L., Yorke, J. A., . . . Salzberg, S. L. (2017). An improved assembly of the loblolly pine mega-genome using long-read single-molecule sequencing. *Gigascience, 6*(1), 1-4. doi:10.1093/gigascience/giw016
