# Supplementary material for: Pinus radiata genome reveals a downward demographic trajectory and opportunities for genomics-assisted breeding
Source: G3 (Bethesda). 2025 Jun 5;15(8):jkaf125. doi: 10.1093/g3journal/jkaf125 (PMC12341877; doi:10.1093/g3journal/jkaf125)
Supplement: jkaf125_Supplementary_Data [file jkaf125_supplementary_data.zip › Table_S2_G3-2024-404909.docx]

**Table S2** Spearman correlation of linkage map marker orders with *P. radiata* pseudomolecules generated by ALLMAPS

| **Pseudomolecule/**  **chromosome**^a^ | **Parent 268405** | **Parent 268345** | **Parent 850055** | **Parent 850096** | **Total chr size (Mbp)** | **Oriented (Mbp)** |
| --- | --- | --- | --- | --- | --- | --- |
| Chr1 | 0.785 | 0.758 | 0.597 | 0.615 | 158.06 | 51.43 |
| Chr2 | 0.976 | 0.880 | 0.853 | 0.420 | 180.22 | 61.14 |
| Chr3 | 0.889 | 0.825 | 0.741 | 0.585 | 149.99 | 41.38 |
| Chr4 | 0.988 | 0.834 | 0.654 | 0.879 | 143.11 | 36.31 |
| Chr5 | 0.920 | 0.784 | 0.675 | 0.374 | 165.53 | 52.16 |
| Chr6 | 0.971 | 0.981 | 0.976 | 0.945 | 133.84 | 30.21 |
| Chr7 | 0.991 | 0.984 | 0.978 | 0.976 | 147.89 | 50.79 |
| Chr8 | 0.964 | 0.900 | 0.705 | NA | 126.38 | 25.12 |
| Chr9 | 0.871 | 0.915 | 0.773 | 0.753 | 168.77 | 45.25 |
| Chr10 | 0.967 | 0.835 | 0.831 | 0.632 | 137.99 | 36.60 |
| Chr11 | 0.981 | 0.954 | 0.888 | 0.259 | 138.94 | 38.39 |
| Chr12 | 0.972 | 0.894 | 0.940 | 0.489 | 139.36 | 39.78 |

^a^Pseudomolecules presumably correspond to chromosomes
