# Supplementary material for: Pinus radiata genome reveals a downward demographic trajectory and opportunities for genomics-assisted breeding
Source: G3 (Bethesda). 2025 Jun 5;15(8):jkaf125. doi: 10.1093/g3journal/jkaf125 (PMC12341877; doi:10.1093/g3journal/jkaf125)
Supplement: jkaf125_Supplementary_Data [file jkaf125_supplementary_data.zip › Table_S3_G3-2024-404909.docx]

**Table S3** Estimated total length of intact retrotransposon elements in the *P. radiata* and *P. taeda* genome assemblies

| **Species** | **Ty3-Gypsy (Mbp)** | **Ty1-Copia**  **(Mbp)** | **LTR (total)**  **(Gbp)** | **Helitron**  **(Mbp)** | **TIR**  **(Mbp)** |
| --- | --- | --- | --- | --- | --- |
| ***Pinus radiata*** | **482** | **302** | **2.25** | **10** | **95** |
| ***Pinus taeda*‡** | **264** | **207** | **0.96** | **3** | **20** |

**‡Zimin et al. (2017)**

Zimin, A. V., Stevens, K. A., Crepeau, M. W., Puiu, D., Wegrzyn, J. L., Yorke, J. A., . . . Salzberg, S. L. (2017). An improved assembly of the loblolly pine mega-genome using long-read single-molecule sequencing. *Gigascience, 6*(1), 1-4. doi:10.1093/gigascience/giw016
