# Supplementary material for: Pinus radiata genome reveals a downward demographic trajectory and opportunities for genomics-assisted breeding
Source: G3 (Bethesda). 2025 Jun 5;15(8):jkaf125. doi: 10.1093/g3journal/jkaf125 (PMC12341877; doi:10.1093/g3journal/jkaf125)
Supplement: jkaf125_Supplementary_Data [file jkaf125_supplementary_data.zip › Table_S4_G3-2024-404909.docx]

**Table S4** Tissues used to generate the *P. radiata* long-read transcriptome dataset. Repeated names are replicates from another sequencing batch.

| **Tissue description** |
| --- |
| Callus |
| Embling |
| Female cone (early) |
| Female cone (late) |
| Male cone (early) |
| Male cone (late) |
| Germinated seed (early) |
| Germinated seed (early) |
| Germinated seed (late) |
| Germinated seed (late) |
| *P. pluvialis* infected needles (field) |
| *P. pluvialis* infected needles (field) |
| *P. pluvialis* infected needles (lab) |
| *P. pluvialis* infected needles (lab) |
| Reproductive bud |
| Reproductive bud |
| Spring xylem |
| Spring xylem |
| Summer xylem |
| Summer xylem |
| Vegetative bud |
| Vegetative bud |
| Phloem |
| Phloem |
