# Supplementary material for: Pinus radiata genome reveals a downward demographic trajectory and opportunities for genomics-assisted breeding
Source: G3 (Bethesda). 2025 Jun 5;15(8):jkaf125. doi: 10.1093/g3journal/jkaf125 (PMC12341877; doi:10.1093/g3journal/jkaf125)
Supplement: jkaf125_Supplementary_Data [file jkaf125_supplementary_data.zip › Table_S5_G3-2024-404909.docx]

**Table S5** Resequencing data: density of segregating sites, nucleotide diversity, Tajima’s *D*, and population scaled recombination rate

| **Category** | **Number** | **Density^a^** | ***π*^b^** | **Tajima's *D*^c^** | **4*N_e_c*^d^** |
| --- | --- | --- | --- | --- | --- |
| Total SNPs | 608,268,251 | 40.5 (21.0,68.2) | 0.0039 (0.0016,0.0086) | –1.34 (–2.11, –0.09) | - |
| Non-singleton SNPs | 432,248,887 | 27.5 (12.2,53.1) | 0.0035 (0.0013,0.0082) | –0.59 (–1.51,0.82) | - |
| Filtered SNPs, MAF ≥ 0.05 | 124,371,054^e^ | - | - | - | 0.00011 (0.00002,0.00162) |
| Filtered SNPs, MAF ≥ 0.10 | 73,943,553^e^ | - | - | - | 0.00008 (0.00001,0.00111) |
| Total indels | 52,343,694 | 3.2 (1.4, 6.5) | - | - | - |

^a^ Average number per kilobase across 10-kb windows (10%, 90% quantile);

^b^ Nucleotide diversity: median across 10-kb windows (10%, 90% quantile);

^c^ Median across 10-kb windows (10%, 90% quantile);

^d^ Scaled recombination rate: median across *N_e_* estimates (10%, 90% quantile);

^e^ Filtering described in Methods
