# Supplementary material for: Pinus radiata genome reveals a downward demographic trajectory and opportunities for genomics-assisted breeding
Source: G3 (Bethesda). 2025 Jun 5;15(8):jkaf125. doi: 10.1093/g3journal/jkaf125 (PMC12341877; doi:10.1093/g3journal/jkaf125)
Supplement: jkaf125_Supplementary_Data [file jkaf125_supplementary_data.zip › Table_S7_G3-2024-404909.docx]

**Table S7** Potential candidate genes on scaffolds with GWAS hits for diameter at breast height. Family/clade/putative ortholog identity based on reciprocal BLAST results against Arabidopsis (Cheng et al., 2017).

| **SNP ID** | **Scaffold** | **Position/s** | **P** | **LD 30 kb^a^** | **Gene Position** | **Family/ Clade/**  **Putative Ortholog** | **Details** |
| --- | --- | --- | --- | --- | --- | --- | --- |
| AX-251856369 | scaffold_28845 | 70665, 85379 | 1.96E-21 | 0.148704219 | 88852..87048 | REDUCED LATERAL ROOT FORMATION (RLF; AT5G09680) putative ortholog | Arabidopsis *RLF* gene encodes a cytosolic protein that positively controls early cell divisions in lateral root initiation but is also involved in shoot organ growth, probably through the control of cell division/proliferation (Ikeyama, Tasaka, & Fukaki, 2010) |
| AX-251590305 | scaffold_30974 | 115331 | 2.29E-13 | 0.177081603 | 2629..3170 | ATP-binding cassette G (ABCG) transporter | Some Arabidopsis ABCG transporters are involved in transport of abscisic acid (ABA) across the plasma membrane and associated with stomatal regulation, others are involved in processes including lignin biosynthesis; ABA has a role in seasonal dormancy (Alejandro et al., 2012; Kang et al., 2010; Kuromori et al., 2010; Tylewicz et al., 2018; Zhang et al., 2021) |
| AX-251279039 | linkedscaffold_3093 | 83307 | 8.45E-10 | 0.215199012 | 126218..129003 | Group C Raf-like protein kinase (MAPKKK family) | Some clade members are involved in ABA signalling and stomatal responses to CO_2_ and red light in Arabidopsis; ABA has a role in seasonal dormancy (Hashimoto et al., 2006; Kamiyama et al., 2021; Matrosova et al., 2015; Tylewicz et al., 2018) |
|  |  |  |  |  | 565340..573426 | PHLOEM INTERCALATED WITH XYLEM-like (PXY/PXL) receptor kinase | Arabidopsis PXY/PXL receptor kinases involved in radial patterning of vascular tissue and secondary growth (Shi, Lebovka, López-Salmerón, Sanchez, & Greb, 2019; Smit et al., 2019; Wang et al., 2019) |
| AX-250517727 | scaffold_170708 | 129261 | 3.28E-08 | 0.155706207 | 129549..132390 | RabA/Rab11 GTPase | Arabidopsis RabA/Rab11 GTPase roles include controlling the cell wall composition in stem tissue (Lunn, Gaddipati, Tucker, & Lycett, 2013) |
|  |  |  |  |  | 145035..145617 | GDSL-type esterase/lipase protein | Arabidopsis homologs have diverse functions including influencing the germination rate and early growth of seedlings and being involved in biotic stress responses (Lai, Huang, Chen, Chan, & Shaw, 2017) |
| AX-251805039 | scaffold_160112 | 32925 | 8.57E-08 | 0.123547589 | 33478..26264 | ATP-binding cassette G (ABCG) transporter | Some Arabidopsis ABCG transporters are involved in transport of abscisic acid (ABA) across the plasma membrane and associated with stomatal regulation, others are involved in processes including lignin biosynthesis; ABA has a role in seasonal dormancy (Alejandro et al., 2012; Kang et al., 2010; Kuromori et al., 2010; Tylewicz et al., 2018; Zhang et al., 2021) |
| AX-251804871 | scaffold_7199 | 33704 | 2.51E-07 | 0.102044365 | 28814..33015 | PIN-FORMED (PIN) auxin efflux carrier family protein | Auxin transporter family, which regulates secondary growth in *Populus* (Zheng et al., 2021). |
| AX-251809478 | linkedscaffold_4564 | 59383 | 5.31E-07 | 0.254824812 | 106968..107335 | PLEIOTROPIC REGULATORY LOCUS (PRL)-type WD40 repeat | Arabidopsis homologs modulate sugar, hormone and stress signalling among other processes (Weihmann, Palma, Nitta, & Li, 2012) |
| AX-251668837 | scaffold_16964 | 176109 | 1.08E-06 | 0.186576921 | 287719..389788 | STRUBBELIG-RECEPTOR FAMILY (SRF) member | Linked to cell wall biology and lignification in Arabidopsis and wood density in *Populus* (Eyüboglu et al., 2007; Porth et al., 2013) |
| AX-251753917 | scaffold_11506 | 44415 | 4.36E-06 | 0.180433232 | 42448..43551 | LEUCINE-RICH REPEAT EXTENSIN (LRX) | Homologs modify cell wall composition and affect cell elongation and plant growth Arabidopsis and half are expressed in wood (predominantly the cambium and the expansion zone) in *Populus* (Abedi, Castilleux, Nibbering, & Niittylä, 2020; Baumberger, Steiner, Ryser, Keller, & Ringli, 2003; Draeger et al., 2015; Zhao et al., 2018) |
| AX-251795808 | scaffold_163321 | 233681 | 5.56E-06 | 0.303371327 | 233896..235406 | YUCCA flavin monooxygenase | *YUCCA* genes encode the rate limiting enzymes in auxin biosynthesis; auxin regulates growth rate and secondary vascular growth |

^a^Average r2 for SNPs up to 30 kb apart for the respective scaffold, >0.2 would indicate long haplotypes in which case we investigated candidate genes on neighbouring scaffolds (denoted ‘linkedscaffold_#’ in column two) as well as the scaffold in which the significant SNP was found.

**References**

Abedi, T., Castilleux, R., Nibbering, P., & Niittylä, T. (2020). The Spatio-Temporal Distribution of Cell Wall-Associated Glycoproteins During Wood Formation in Populus. *Frontiers in Plant Science, 11*. doi:10.3389/fpls.2020.611607

Alejandro, S., Lee, Y., Tohge, T., Sudre, D., Osorio, S., Park, J., . . . Martinoia, E. (2012). AtABCG29 is a monolignol transporter involved in lignin biosynthesis. *Current Biology, 22*(13), 1207-1212. doi:<https://doi.org/10.1016/j.cub.2012.04.064>

Baumberger, N., Steiner, M., Ryser, U., Keller, B., & Ringli, C. (2003). Synergistic interaction of the two paralogous Arabidopsis genes *LRX1* and *LRX2* in cell wall formation during root hair development. *The Plant Journal, 35*(1), 71-81. doi:<https://doi.org/10.1046/j.1365-313X.2003.01784.x>

Cheng, C.-Y., Krishnakumar, V., Chan, A. P., Thibaud-Nissen, F., Schobel, S., & Town, C. D. (2017). Araport11: a complete reannotation of the *Arabidopsis thaliana* reference genome. *The Plant Journal, 89*(4), 789-804. doi:<https://doi.org/10.1111/tpj.13415>

Draeger, C., Ndinyanka Fabrice, T., Gineau, E., Mouille, G., Kuhn, B. M., Moller, I., . . . Ringli, C. (2015). Arabidopsis leucine-rich repeat extensin (LRX) proteins modify cell wall composition and influence plant growth. *BMC Plant Biology, 15*(1), 155. doi:10.1186/s12870-015-0548-8

Eyüboglu, B., Pfister, K., Haberer, G., Chevalier, D., Fuchs, A., Mayer, K. F. X., & Schneitz, K. (2007). Molecular characterisation of the STRUBBELIG-RECEPTOR FAMILY of genes encoding putative leucine-rich repeat receptor-like kinases in Arabidopsis thaliana. *BMC Plant Biology, 7*(1), 16. doi:10.1186/1471-2229-7-16

Hashimoto, M., Negi, J., Young, J., Israelsson, M., Schroeder, J. I., & Iba, K. (2006). Arabidopsis HT1 kinase controls stomatal movements in response to CO_2_. *Nature Cell Biology, 8*(4), 391-397. doi:<http://www.nature.com/ncb/journal/v8/n4/suppinfo/ncb1387_S1.html>

Ikeyama, Y., Tasaka, M., & Fukaki, H. (2010). RLF, a cytochrome b_5_-like heme/steroid binding domain protein, controls lateral root formation independently of ARF7/19-mediated auxin signaling in *Arabidopsis thaliana*. *The Plant Journal, 62*(5), 865-875. doi:<https://doi.org/10.1111/j.1365-313X.2010.04199.x>

Kamiyama, Y., Hirotani, M., Ishikawa, S., Minegishi, F., Katagiri, S., Rogan, C. J., . . . Umezawa, T. (2021). Arabidopsis group C Raf-like protein kinases negatively regulate abscisic acid signaling and are direct substrates of SnRK2. *Proceedings of the National Academy of Sciences of the United States of America, 118*(30), e2100073118. doi:doi:10.1073/pnas.2100073118

Kang, J., Hwang, J.-U., Lee, M., Kim, Y.-Y., Assmann, S. M., Martinoia, E., & Lee, Y. (2010). PDR-type ABC transporter mediates cellular uptake of the phytohormone abscisic acid. *Proceedings of the National Academy of Sciences of the United States of America, 107*(5), 2355-2360. doi:10.1073/pnas.0909222107

Kuromori, T., Miyaji, T., Yabuuchi, H., Shimizu, H., Sugimoto, E., Kamiya, A., . . . Shinozaki, K. (2010). ABC transporter AtABCG25 is involved in abscisic acid transport and responses. *Proceedings of the National Academy of Sciences of the United States of America, 107*(5), 2361-2366. doi:10.1073/pnas.0912516107

Lai, C.-P., Huang, L.-M., Chen, L.-F. O., Chan, M.-T., & Shaw, J.-F. (2017). Genome-wide analysis of GDSL-type esterases/lipases in Arabidopsis. *Plant Molecular Biology, 95*(1), 181-197. doi:10.1007/s11103-017-0648-y

Lunn, D., Gaddipati, S. R., Tucker, G. A., & Lycett, G. W. (2013). Null mutants of individual RABA genes impact the proportion of different cell wall components in stem tissue of *Arabidopsis thaliana*. *PLoS One, 8*(10), e75724. doi:10.1371/journal.pone.0075724

Matrosova, A., Bogireddi, H., Mateo-Peñas, A., Hashimoto-Sugimoto, M., Iba, K., Schroeder, J. I., & Israelsson-Nordström, M. (2015). The HT1 protein kinase is essential for red light-induced stomatal opening and genetically interacts with OST1 in red light and CO_2_-induced stomatal movement responses. *New Phytologist, 208*(4), 1126-1137. doi:10.1111/nph.13566

Porth, I., Klapšte, J., Skyba, O., Hannemann, J., McKown, A. D., Guy, R. D., . . . Mansfield, S. D. (2013). Genome-wide association mapping for wood characteristics in *Populus* identifies an array of candidate single nucleotide polymorphisms. *New Phytologist, 200*(3), 710-726. doi:<https://doi.org/10.1111/nph.12422>

Shi, D., Lebovka, I., López-Salmerón, V., Sanchez, P., & Greb, T. (2019). Bifacial cambium stem cells generate xylem and phloem during radial plant growth. *Development, 146*(1). doi:10.1242/dev.171355

Smit, M. E., McGregor, S. R., Sun, H., Gough, C., Bågman, A.-M., Soyars, C. L., . . . Etchells, J. P. (2019). A PXY-mediated transcriptional network integrates signaling mechanisms to control vascular development in Arabidopsis. *The Plant Cell, 32*(2), 319-335. doi:10.1105/tpc.19.00562

Tylewicz, S., Petterle, A., Marttila, S., Miskolczi, P., Azeez, A., Singh, R. K., . . . Bhalerao, R. P. (2018). Photoperiodic control of seasonal growth is mediated by ABA acting on cell-cell communication. *Science, 360*(6385), 212-215. doi:doi:10.1126/science.aan8576

Wang, N., Bagdassarian, K. S., Doherty, R. E., Kroon, J. T., Connor, K. A., Wang, X. Y., . . . Etchells, J. P. (2019). Organ-specific genetic interactions between paralogues of the PXY and ER receptor kinases enforce radial patterning in Arabidopsis vascular tissue. *Development, 146*(10). doi:10.1242/dev.177105

Weihmann, T., Palma, K., Nitta, Y., & Li, X. (2012). *PLEIOTROPIC REGULATORY LOCUS 2* exhibits unequal genetic redundancy with its homolog *PRL1*. *Plant and Cell Physiology, 53*(9), 1617-1626. doi:10.1093/pcp/pcs103

Zhang, Y., Vasuki, H., Liu, J., Bar, H., Lazary, S., Egbaria, A., . . . Shani, E. (2021). ABA homeostasis and long-distance translocation is redundantly regulated by ABCG ABA importers. *bioRxiv*, 2021.2005.2012.443788. doi:10.1101/2021.05.12.443788

Zhao, C., Zayed, O., Yu, Z., Jiang, W., Zhu, P., Hsu, C.-C., . . . Zhu, J.-K. (2018). Leucine-rich repeat extensin proteins regulate plant salt tolerance in *Arabidopsis*. *Proceedings of the National Academy of Sciences of the United States of America, 115*(51), 13123-13128. doi:doi:10.1073/pnas.1816991115

Zheng, S., He, J., Lin, Z., Zhu, Y., Sun, J., & Li, L. (2021). Two MADS-box genes regulate vascular cambium activity and secondary growth by modulating auxin homeostasis in *Populus*. *Plant Communications, 2*(5), 100134. doi:<https://doi.org/10.1016/j.xplc.2020.100134>
